# Factors associated with the uptake of long-acting reversible contraception among female sex workers in post-conflict Northern Uganda: a cross-sectional study

**DOI:** 10.1186/s12978-022-01345-6

**Published:** 2022-02-02

**Authors:** Simple Ouma, Nazarius Mbona Tumwesigye, Catherine Abbo, Rawlance Ndejjo

**Affiliations:** 1 Department of Research, The AIDS Support Organization (TASO), Kampala, Uganda; 2 Department of Epidemiology and Biostatistics, School of Public Health, College of Health Sciences, Makerere University, Kampala, Uganda; 3 Department of Psychiatry, School of Medicine, College of Health Sciences, Makerere University, Kampala, Uganda; 4 Department of Disease Control and Environmental Health, School of Public Health, College of Health Sciences, Makerere University, Kampala, Uganda

**Keywords:** Female sex workers, Long-acting reversible contraception, Post-conflict setting, Northern Uganda

## Abstract

**Background:**

Long-acting reversible contraception (LARC) is the most effective and reliable contraception option for female sex workers (FSWs) who desire future fertility. Unlike the other reversible contraceptive methods, LARC use requires only periodic users’ involvement at the time of application and re-application. However, only a few studies on LARC uptake among FSWs are available in Uganda. To fill this knowledge gap, we examined factors associated with the uptake of LARC among FSWs in post-conflict Northern Uganda.

**Methods:**

We conducted a cross-sectional study among adult FSWs operating in the post-conflict Gulu district in Northern Uganda. We collected quantitative data among 280 FSWs of reproductive ages (18–49 years) who were neither pregnant nor using permanent contraception. We utilized a pretested semi-structured questionnaire to gather information from each participant through face-to-face interviews. We collected data on socio-demographic characteristics, sex work-related characteristics, obstetric history, HIV status, and LARC uptake. Data were then entered into EPI INFO 7, cleaned, and analyzed using multivariable Poisson regression in STATA 14.0 to obtained adjusted prevalence ratios (PR).

**Results:**

Among the study participants: the mean age (SD, range) was 26.5 (5.9, 18–45) years, 48.6% reported at least one unintended pregnancy during sex work, and 37.4% had at least one induced abortion. Meanwhile, only less than two in three (58.6%) participants were using LARC. At multivariable level, factors that remained independently associated with LARC uptake included: longer duration of sex work (adjusted PR = 1.44, 95% CI: 1.03–2.02), higher parity (adjusted PR = 1.13, 95% CI: 1.02–1.26), history of unintended pregnancies during sex work (adjusted PR = 1.24 CI: 1.01–1.51), and being a brothel/lodge-based FSWs (adjusted PR = 1.28, 95% CI: 1.01–1.63).

**Conclusions:**

The above findings revealed a gap in the uptake of LARC among FSWs in post-conflict Northern Uganda influenced by duration of sex work, parity, unintended pregnancies during sex work, and place of sex work. Interventions to improve LARC uptake should target the newly recruited FSWs with low parity and the non-brothel/lodge-based FSWs.

## Plain language summary

Long-acting reversible contraception (LARC) is the most effective and reliable family planning method for female sex workers (FSWs) who desire future fertility. LARC is very reliable since it requires only periodic users' involvement at the time of application or re-application. However, only a few studies on the uptake of LARC among FSWs are available in Uganda.

To fill the above knowledge gap, we examined the factors associated with the uptake of LARC among the FSWs in post-conflict Northern Uganda. We collected cross-sectional quantitative data among 280 non-pregnant FSWs of reproductive age (18–49 years) who were not on a permanent family planning method. We analyzed the data using Poisson regression to investigate possible associations between uptake of LARC and the various independent factors.

The results showed that uptake of LARC was more common among participants who had been sex workers for a longer period, had more children, experienced unintended pregnancies during sex work, and was doing sex work from brothels or lodges. The findings revealed a gap in the uptake of LARC among FSWs in post-conflict Northern Uganda. Therefore, to increase LARC uptake among FSWs, future family planning interventions should target the newly recruited FSWs, FSWs with few children, and FSWs who do not work in brothels or lodges.

## Background

In 2019, up to 49% of all pregnancies in the low- and middle-income countries (LMICs) were unintended [1]. This high magnitude of unintended pregnancy in the LMICs poses a public health challenge to the national healthcare systems. Among the female sex workers (FSWs) in the LMICs, unintended pregnancy is a common phenomenon with a very high rate of 27.1 per 100 person-years [2]. However, most of these unintended pregnancies among the FSWs result in induced abortions that lead to severe and often life-threatening post-abortion complications [3–5]. Having numerous sexual partners, high frequencies of intercourse and high levels of stigma are the key drivers of unintended pregnancy among FSWs [2]. In addition, sexual gender-based violence also increases the risk of unintended pregnancy in this population [6].

Previous multi-country research in the LMICs revealed suboptimal uptakes of long-acting reversible contraception (LARC) among the FSWs [7]. In Uganda, multiple studies reported very high rates of unintended pregnancies among FSWs [5, 8–10]- an indirect indicator of the high unmet need for contraception in this underserved population. Instead of LARC, FSWs of reproductive age tend to use the less reliable short-acting reversible contraceptives, especially condom [11]. Yet, condom use does not provide complete protection against pregnancy among the FSWs due to the rampant inconsistency of condom use along with condom breakage in this population [12–18]. In Uganda, the uptake of LARC is suboptimal in women in the general population [19], which trend could be more pronounced among FSWs. The single biggest challenge in reducing the high rate of unintended pregnancy among FSWs in Uganda is the lack of dedicated family planning programs for this underserved population. Currently, most healthcare programs mainly target HIV services and only dedicate little resources for family planning services [12–14, 20].

Family planning programmers need to increase the proportions of FSWs who use the more effective and reliable LARC to fulfill their contraception needs [21–25], especially in conflict-affected settings. Increasing the uptake of LARC among FSWs in post-conflict settings requires a good understanding of the predictors of LARC uptake, which remains understudied [26]. Research on LARC uptake among the FSWs in Uganda could have been affected by the illegal nature of sex work in the country [27]. Increasing the uptake of LARC would help reduce the high rates of unintended pregnancy and induced abortion among FSWs and thus improving their overall sexual and reproductive health [28]. This study aimed at determining factors associated with LARC uptake among FSWs operating in post-conflict Northern Uganda. The study findings will generate the evidence needed by the ministry of health and other stakeholders to inform appropriate interventions to promote LARC uptake among FSWs.

## Methods

### The study aim, design, setting, and population

The study aimed to determine the associated factors of LARC uptake among FSWs in post-conflict Northern Uganda. We conducted a cross-sectional study to collect quantitative data among adult FSWs in the post-conflict Gulu district in Northern Uganda. People living in Northern Uganda are still undergoing economic recovery from the more than 20 years of the Lord’s Resistance Army rebellion that devastated social and economic livelihoods in the region. Currently, more than 80% of people in Gulu are subsistence farmers [29]. Gulu district is a regional business hub in Northern Uganda with an estimated 1425 FSWs who operate in the district [30]. Much as most FSWs in Gulu are mobile, the majority live in Gulu city. Most FSWs in the region get their HIV preventive and treatment services from The AIDS Support Organization (TASO). TASO is a national Non-Governmental Organization that provides comprehensive HIV services across Uganda [31]. TASO is one of the few organizations that provide HIV prevention, care, and treatment services to FSWs in the country. Thus, TASO Gulu conducts regular mapping of FSWs in the region. Recently, TASO Gulu mapped more than 1300 FSWs in the Gulu.

### Sample size and sampling

We calculated a sample size of 380 FSWs using the Cochran formula (1963, 1975) [32] as part of a project that studied depression among FSWs in the district. We published the detail of the sample size calculation elsewhere [33]. Our sample included all the FSWs aged 18 years and above who had been active in sex work within 6 months before data collection. We identified a total of 789 adult FSWs from the database at TASO Gulu. We utilised an online random number generator to select 380 participants by simple random sampling technique. To minimize non-response bias, we reached out to the selected FSWs through phone calls, through peers, physically traced them using the mapping information at TASO with details of sex work venues, hang-out places, and residence of FSWs, or met them during clinic days. Out of the 380 selected FSWs, we successfully tracked 302 participants, of whom; two declined to consent and 20 excluded as guided by the exclusion criteria. Those excluded were not eligible to use any modern contraception: 17 were pregnant, two underwent bilateral tubal ligation, and one was post- menopausal. Thus, the remaining 280 participants responded to the questionnaire at the conducive places they chose.

### Data collection and management

From 20th March to 2nd June 2020, we conducted face-to-face interviews to gather information from participants. However, before data collection, we pretested the semi-structured questionnaire developed in English and later translated into the local language of Acholi. The pretesting of the questionnaire was conducted among 20 FSWs from the neighboring Amuru district to check for consistency in question interpretation and language appropriateness. Together with a female research assistant, the first author collected participant’s data in either Acholi or English as dictated by participant’s literacy level and preference. Independent variables in this study included: socio-demographic characteristics, sex work-related characteristics, monthly income, place of sex work, sex work-related mobility, alcohol use, HIV status, and obstetric histories. The only outcome variable was the uptake of LARC.

### Statistical analysis

Data were entered and cleaned using EPI INFO 7 and exported to STATA 14.0 for analysis. We presented univariate results using frequencies with corresponding proportions or means with corresponding standard deviations (SD) as appropriate. To examine associations between LARC uptake and independent variables, we computed the prevalence ratios (PR) using bivariable Poisson regression with robust variances instead of the odds ratios to avoid overestimating the effect size [34, 35]. We presented results from the bivariable analyses using the unadjusted PR with their corresponding 95% confidence intervals (CI) and *p*-values. Before running the multivariable Poisson regression, we first checked the significant variables (*p* < *0.05*) for multicollinearity. When two or more variables showed multicollinearity (r ≥ 0.4), we included only one in the final model. Thus, we excluded both age (r = 0.44) and marital status (r = 0.42) from the multivariable model after showing correlations with parity. Likewise, we excluded both history of induced abortion (r = 0.60) and lifetime unplanned pregnancy (0.78) from the multivariable model after showing correlations with unplanned pregnancy during sex work. Finally, we added all the remaining variables in the multivariable Poisson regression model and executed a stepwise backward elimination technique until a “best fit” model was derived. Predictors from bivariable and multivariate analyses whose CI did not include the null value (1.0) were considered significant.

## Results

### Socio-demographic, obstetric, and sex work-related characteristics of participants

Among the study participants: the mean age (SD, range) was 26.5 (± 5.9, 18–45) years, more than two-thirds (67.9%) were below 30 years of age, and 60.7% had either primary or no education. Almost one-third (31.6%) of the respondents had an average monthly income within the first income quartile (< $28), 53.2% had never been married, 92.6% had at least one previous pregnancy, and 48.9% had at least one unintended pregnancy during sex work. While 95.4% used condoms during their last sexual encounters, only two-thirds (66.1%) reported using condoms consistently (Table 1).

**Table 1:** Demographic and reproductive health characteristics of the participants

Characteristic	Number (N.)	Percentage (%)
Age (in completed years)		
< 30	190	67.9
≥ 30	90	32.1
Education level		
≤ Primary education	170	60.7
> Primary education	110	39.3
Marital status		
Never married	149	53.2
Cohabiting/Married	36	12.9
Divorced	75	26.8
Widowed	20	7.1
Parity		
0–1	120	42.9
2 +	160	57.1
History of unintended pregnancy during sex work		
No	144	51.4
Yes	136	48.6
History of unintended pregnancy in her lifetime		
No	115	41.1
Yes	165	58.9
History of induced abortion		
No	176	63.1
Yes	1.03	36.9
Had post-abortion care		
No	233	83.2
Yes	47	16.8
Duration as a sex worker (completed years)		
≤ 1	57	20.4
2 +	223	79.6
Average monthly income ($)		
1st quartile (< 28)	88	31.6
2nd quartile (28–54)	74	26.5
3rd quartile (55–95)	52	18.6
4th quartile (> 95)	65	23.3
Used condoms consistently		
No	95	33.9
Yes	185	66.1
Work from lodges/brothel		
No	91	32.5
Yes	189	67.5
Raped during sex work		
No	221	78.9
Yes	59	21.1
HIV-positive		
No	164	58.8
Yes	115	41.2

### Uptake of LARC among FSWs in Gulu district

**Fig. 1:**
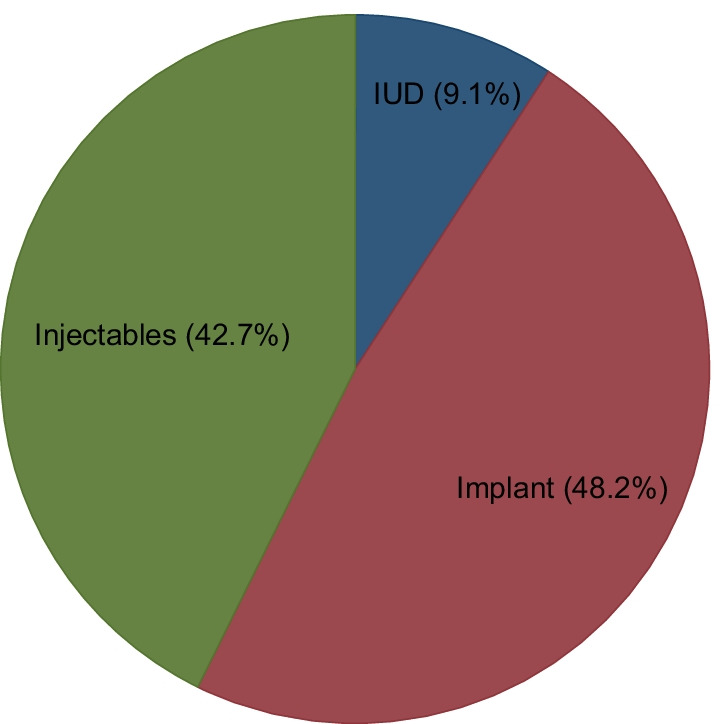
Showing uptake of LARC among FSWs in post-conflict Gulu district

Less than two-thirds (58.6%) of the participants were using LARC. In this population, the most commonly used LARC options were the Implants (48.2%) and the injectables (42.7%). Meanwhile, the least utilised LARC option was the IUD (9.1%) (Fig. 1).

### Factors associated with LARC uptake among FSWs in Gulu district

The results from the bivariate analyses revealed several factors with positive associations with the uptake of LARC. These factors were: older age (*p* = *0.048*), higher parity [≥ two] (*p* = *0.001*), being married/cohabiting (*p* = *0.021*), history of induced abortions (*p* = *0.011*), history of post-abortion care (*p* < *0.001*), and history of lifetime unplanned pregnancy (*p* = *0.002*). The other associated factors were: history of unplanned pregnancy during sex work (*p* = *0.003*), longer duration (≥ 2 years) of sex work (*p* < *0.001*). In addition, higher monthly income falling within the 4th quartile (*p* = *0.044*), mobile sex work (*p* = *0.023*), living with HIV (*p* = *0.027*), and being a brothel/lodge-based sex work (*p* = *0.004*) also influenced LARC uptake. However, at the multivariable level, the only factors that remained independently associated with LARC uptake were: longer duration of sex work [≥ 2 years] (adjusted PR = 1.44, 95% CI: 1.03–2.02), higher parity [≥ two] (adjusted PR = 1.13, 95% CI: 1.02–1.26), history of unintended pregnancy during sex work (adjusted PR = 1.24 CI: 1.01–1.51), and being a brothel/lodge-based sex worker (adjusted PR = 1.28, 95% CI: 1.01–1.63) (Table 2).

**Table 2:** Factors associated with the uptake of LARC among FSWs in Gulu

Factor	Using LARC Yes No		Unadjusted PR (95%CI)	Adjusted PR (95%CI)
	N (%)	N (%)	Bivariate Model Multivariable Model	
Age (years)				
< 30	104 (54.7)	86 (45.3)	1.00	–
≥ 30	60 (66.7)	30 (33.3)	1.22 (1.002–1.48) *	–
Marital status				
Single	80 (53.7)	69(46.3)	1.00	–
Married/cohabiting	26 (72.2)	10(27.8)	1.35 (1.05–1.73) *	–
Divorced	48 (64.0)	27 (36.6)	1.19 (0.95–1.49)	–
Widow	10 (50.0)	10 (50.0)	0.93 (0.59–1.48)	–
Parity				
0–1	64 (53.3)	56 (46.7)	1.00	1.00
2 +	52 (32.5)	108 (67.5)	1.45 (1.16–1.80) **	1.13 (1.02–1.26) *
Had induced abortion				
No	93 (52.8)	83 (47.2)	1.00	–
Yes	70 (68.0)	33 (32.0)	1.29 (1.06–1.56) *	–
Had post-abortion care				
No	127 (54.5)	106 (45.5)	1.00	–
Yes	37 (78.7)	10 (21.3)	1.44 (1.19–1.75) ***	–
Had unintended pregnancy				
No	54 (47.0.2)	61 (53.0)	1.00	–
Yes	110 (66.7)	55 (33.3)	1.42 (1.14–1.77) **	–
Unintended pregnancy during sex work				
No	72 (50.0)	72 (50.0)	1.00	1.00
Yes	92 (67.7)	44 (32.3)	1.35 (1.11–1.65) **	1.24 (1.01–1.51) *
Duration of sex work				
≤ one year	22 (38.6)	35 (61.4)	1.00	1.00
2 + years	142(63.7)	81 (36.3)	1.65 (1.17–2.32) **	1.44 (1.03–2.02) *
Average monthly income				
1st quartile	44 (50.0)	44 (50.0)	1.00	–
2nd quartile	42 (56.8)	32 (43.2)	1.14 (0.85–1.52)	–
3rd quartile	34 (65.4)	18 (34.6)	1.31 (0.98–1.74)	–
4th quartile	43 (66.1)	22 (33.9)	1.32 (1.01–1.74) *	–
Nature of sex work				
Urban only	69 (51.9)	64 (48.1)	1.00	–
Rural only	10 (55.6)	8 (44.4)	1.07 (0.69–1.67)	–
Mobile	85 (65.9)	44 (34.1)	1.27 (1.03–1.56) **	–
HIV-status				
HIV-Negative	87 (53.0)	77 (47.0)	1.00	–
HIV-Positive	76 (66.1)	39 (33.9)	1.25 (1.03–1.51) *	–
Lodges/Brothels-based				
No	41 (45.0)	50 (55.0)	1.00	–
Yes	123 (65.1)	66 (34.9)	1.44 (1.12–1.86) **	1.28 (1.01–1.63) *

*p < 0.05, **p < 0.01, ***p < 0.001

## Discussion

This study examined LARC uptake among FSWs in post-conflict Gulu, Northern Uganda. Less than two-thirds (58.6%) of FSWs were using LARC. Among the LARC users, the majority were either on Implants (48.2%) or injectables (42.7%) and only (9.1%) were on IUDs. Independent factors associated with utilization of LARC were: longer duration of sex work (≥ 2 years), higher parity (≥ two), history of unintended pregnancy during sex work, and being a brothel/lodge-based sex worker. In this study, the prevalence of LARC stood at 58.6%. Compared to this study, higher proportions of FSWs were using the LARC in Zambia [66.6%], Kenya [64.6%], and Ethiopia [69.2%] [36–38]. The observed level of LARC uptake is suboptimal, yet only two-thirds of FSWs were consistently using condoms. Many women in Nothern Uganda mainly rely on condoms for contraception while avoiding the LARC because of their desire to have many children [39]. However, the reliability of condoms to prevent pregnancies among FSWs is very weak- nearly half (47.5%) of FSWs experience condom failure [40] in 1 month [36]. Among the users of LARC, the majority were on Implant (48.2%) and injectables (42.7%). The finding agrees with the reported trend in LARC uptake among the general population of women of reproductive age in the region [39].

Results indicate that LARC uptake was 28% higher among brothel/lodge-based FSWs. This finding is in line with a report from HIV prevention programs targeting FSWs in developing countries showing that FSWs in brothels or lodges were more likely to use LARC since they are more organized and have easier access to health care services [41]. Therefore, to increase access to LARC, future family planning interventions for the FSWs should dedicate more resources to target the FSWs operating in isolated hotspots other than the brothels or lodges. In addition, FSWs who had worked longer as sex workers had a 44% increase in the utilization of LARC compared to their counterparts who had worked as sex workers for shorter periods. This finding is contrary to observations among FSWs in China and Russia, where a longer duration of sex work did not affect LARC uptake [15, 16]. However, it is worth noting that in Uganda, most of the newer FSWs are adolescents and young women who have restricted access to modern contraceptives. In fact, in this study, we found that the older FSWs with two or more children had a higher prevalence of LARC use. Moreover, FSWs in China and Russia could be having higher levels of LARC awareness, a better attitude towards LARC use, and desires for smaller numbers of children as dictated by policies like the one-child policy in China.

Furthermore, FSWs who experienced unintended pregnancies during sex work were 24% more likely to use LARC. These subgroups of FSWs take up LARC to prevent future unintended pregnancies. Using LARC saves money and inconveniences since FSWs were more likely to terminate most of the previous unintended pregnancies during sex work (*p* < *0.001*). This finding agrees with a report showing that FSWs who had terminated unintended pregnancies during sex work were more likely to use LARC [15, 42]. The increased likelihood of LARC uptake among FSWs with induced abortion is because women who ever had induced abortion may use LARC to avoid the bad experiences of pregnancy [43, 44]. Lastly, FSWs with higher parity [≥ two] had a 22% increase in LARC uptake. The finding is in agreement with previous reports among FSWs in Swaziland [12], India [45], and Tanzania [46]. The low uptake of LARC among women with lower parity is in line with a previous report showing that most women (93.4%) in Northern Uganda desire to have at least three children [39]. Therefore, the desire for future fertility may make FSWs with lower parity avoid using LARC. Therefore, there is a need for sensitization on LARC among the younger FSWs with low parity.

### Strengths and limitations of this study

Unlike most previous studies that used non-probability sampling methods among FSWs, we selected a representative sample of FSWs using a random sampling technique. Thus, current findings are more generalizable to similar contexts. However, the study had some limitations. We conducted a cross-sectional study that elicited associations but not causation. Secondly, the information collected may have been influenced by recall bias since we asked FSWs about their past. Lastly, some of the implored information relating to sex work were sensitive and difficult to provide. However, the interviewers had close working relationships with the FSWs.

## Conclusions

The findings revealed a gap in the uptake of LARC among FSWs in post-conflict Northern Uganda influenced by duration of sex work, parity, sex work-related unintended pregnancies, and place of sex work. Interventions to improve LARC uptake should target; the newly recruited FSWs, FSWs with low parity, and the non-brothel/lodge-based FSWs.

## Data Availability

The datasets used and analyzed during the current study are available from the corresponding author on reasonable request to oumasimple@gmail.com.

## Abbreviations

CI: Confidence interval
FSWs: Female sex workers
HIV: Human immunodeficiency virus
LARC: Long-acting reversible contraception
LMICs: Low- and middle-income countries
PR: Prevalence ratio
SD: Standard deviation
TASO: The AIDS support organization
